# An Innovative Population Health Tool for Overall Health Status Assessment: Prospective Observational Study

**DOI:** 10.2196/74101

**Published:** 2026-01-26

**Authors:** Bibina Tuty Umaira Hj Abd Hamid, Ronald Wihal Oei, Norhayati Kassim, Ryutaro Oikawa, Norzawani Ishak, Si Yee Chan, Jane Tey, Pijika Watcharapichat, Joshua Lam, Pg Dr Noor Azmi Mohammad

**Affiliations:** 1 Ministry of Health, Bandar Seri Begawan, Brunei Darussalam, Bandar Seri Begawan, Brunei Darussalam; 2 Department of Medical Advisory, EVYD Technology Sdn Bhd, Brunei Darussalam, RBPC 12, Simpang 265-233-185, Jalan Jerudong, Kg Jerudong, Mukim Sengkurong, BG3122, Brunei Darussalam, 673 69103818

**Keywords:** population health tool, overall health assessment, digital health application, chronic diseases, health score

## Abstract

**Background:**

The World Health Organization reported that noncommunicable diseases (NCDs) contribute to around 74% of deaths worldwide. A similar phenomenon can also be observed in Brunei Darussalam. One of the most cost-effective approaches to control the growing burden of NCDs is to reduce related modifiable risk factors.

**Objective:**

This study aims to propose a composite health score called Health Index, inspired by the 6 pillars of lifestyle medicine, which acts as a measure of health and can show how health changes over time at an individual and national level.

**Methods:**

Health Index is a series of questionnaires that captures users’ health status on several domains of health and, upon completion, the users are categorized as either healthy, at risk, or in poor health. Users will also be able to view health advice based on their answers to the questionnaires.

**Results:**

The field testing results show Health Index as a promising population health management tool. 13.8% (166/1200) of the targeted users completed Health Index within 1 month, with 85% (1019/1200) of them in the “At Risk” category. We also identified diet as the most prominent health issue.

**Conclusions:**

In conclusion, the Health Index potentially enables early detection and management of NCD risk factors to mitigate the high cost of advanced disease and complications. In the future, we aim to retrospectively and prospectively validate the Health Index through several statistical analyses.

## Introduction

### Background

Noncommunicable diseases (NCDs) are a diverse group of chronic conditions that are nontransmissible. They typically develop over a long duration and are often the result of a combination of genetic, physiological, environmental, and behavioral factors. There are 4 main diseases that are dominant in NCDs’ mortality and morbidity, which are cardiovascular diseases (CVDs), cancer, chronic respiratory diseases, and diabetes. NCDs are now the leading cause of death globally, posing a major challenge to public health and sustainable development.

According to the World Health Organization (WHO), 74% of deaths are due to NCDs worldwide [1]. Each year, around 17 million individuals aged 70 years and below die prematurely due to NCDs [2]. CVDs account for most NCD deaths by contributing around 17.9 million deaths annually. Subsequently, cancers contribute to 9.3 million deaths, chronic respiratory diseases to 4.1 million, and diabetes to 2 million. Similarly, NCDs, including CVDs, diabetes mellitus, and cancer, are major causes of death in Brunei Darussalam [2]. In 2021, the top 5 NCDs that led to death and disability (disability-adjusted life years) in Brunei Darussalam were ischemic heart disease, stroke, diabetes, chronic kidney disease, and lung cancer [3]. In 2019, the World Health Assembly called for the development of an Implementation Roadmap 2023 to 2030, which aims at reducing one-third of premature deaths from NCDs by 2030 [4]. While these statistics provide an important overview of NCD burden, they must be interpreted with caution. Global estimates from WHO are often based on modeled data and may be influenced by limitations in data collection, particularly in low- and middle-income countries where health surveillance systems may be less robust. Similarly, national data from Brunei Darussalam may face challenges related to the completeness of health records and time lags in reporting.

Most NCDs are the result of several factors, including genetic, environmental risk factors, metabolic risk factors, and modifiable behavioral risk factors [2]. The latter includes smoking, low physical activity, unhealthy diet, and excessive consumption of alcohol, all of which increase the risk of NCDs. The top metabolic and behavioral risk factors that contribute to disability-adjusted life years in Brunei Darussalam include high fasting plasma glucose, high BMI, an unhealthy diet, smoking, and high blood pressure [5]. Hence, the majority of NCDs are preventable, as their onset typically stems from lifestyle factors early in life. Therefore, reducing the major modifiable risk factors is the most cost-effective way to control the growing burden of NCDs. Despite being preventable, efforts to reduce these modifiable risk factors have seen limited global success. One of the main barriers is the general lack of awareness and understanding of NCDs and their risk factors among both the public and policymakers. This underscores the pressing need to enhance education and public health communication. Technology, including digital health platforms, can support these efforts by enabling scalable, personalized, and timely dissemination of health information and empowering individuals to take proactive steps toward healthier lifestyles [6].

Given the growing burden of NCDs and the importance of early risk identification, there is a need for accessible, evidence-based tools that can support population-level NCD prevention efforts. One concept that has been becoming increasingly popular is the use of a Health Score [7-11]. It quantifies an individual’s overall health condition comprehensively as a number. It factors in multiple aspects of health, such as physical health, physical activity, diet, and others. The health score is the result of integrating various data points into a single, actionable metric. Key limitations of current health scores are limited health domain coverage and limited downstream applications.

Lifestyle medicine is a field of medicine that applies therapeutic lifestyle interventions as the primary approach for managing chronic conditions, such as CVDs, type 2 diabetes, obesity, and others. There are 6 pillars of lifestyle medicine, which are mental well-being, healthy relationships, physical activity, healthy eating, sleep, and minimizing harmful substances.

Given the limitations of the existing health scores and based on the lifestyle medicine concept, we propose a composite health score called Health Index, which acts as a measure of health and can show how health changes over time at the individual and national level. Health Index addresses some of the major limitations of existing health scores. Health Index covers 5 important health domains, which are inspired by the pillars of lifestyle medicine. Furthermore, Health Index supports a broader range of downstream applications and also health care system connectivity. This initiative comes from a partnership between the Brunei Darussalam Ministry of Health and EVYD Technology Sdn Bhd. The Health Index score is calculated based on users’ health status in several domains of health. It is developed as a dynamic digital solution that updates the user’s health status based on data from health records and self-reported information that will be available on BruHealth (EVYD Technology Sdn Bhd).

BruHealth is Brunei Darussalam’s national mobile health app (mHealth app) launched in May 2020 as a response to the COVID-19 pandemic to assist with contact tracing. The app was later expanded during the rollout of the National COVID-19 vaccination as it was used as a platform for booking vaccination appointments. It has been reported that about 4,37,711 users have registered with BruHealth [12]. Over time, as the pandemic responses were scaled down, BruHealth has evolved into a platform for health promotion programs and health management plans.

### Problem Statement

So far, there is no scientifically proven single set of measures that can fully describe the health of a large and diverse population. Most measures only scrutinize one aspect of health, for example, cardiovascular risk [13], kidney health [14], and cancer [15,16]. These measures are not ideal when assessing the overall health status of individuals or a population.

The Centers for Disease Control and Prevention (CDC) and other global health agencies often consider life expectancy and mortality data to assess overall population health. However, it is worth noting that these metrics do not consider different aspects of health. Therefore, actionable insights cannot be obtained from such metrics. Pano et al [7] proposed a health score derived from a combination of sociodemographic, anthropometric, and lifestyle data, called “Lifestyle and Well-Being Index (LWB-I),” which takes into consideration 12 lifestyle-related features. However, several limitations exist in the proposed index. First, all the features are self-reported and do not take into account physical health-related variables, such as biomarker levels and early metabolic changes. Second, the index was evaluated only on the young adult population who were followed up for 4 years. The evaluation process excluded other age groups, such as the older population, who are more at risk of developing chronic diseases and poor health. There are also several commercially developed health scores available, such as Sprout [8], ConnectedLife [9], Aktivo Score [10], Dacadoo [11], and others. Table 1 summarizes the differences between these products and our proposed Health Index.

**Table 1.** Summary of commercially available health scores and comparison with Health Index.

Aspects	Sprout [8]	ConnectedLife [9]	Aktivo score [10]	Dacadoo [11]	Health Index
Country	Canada	Singapore	Singapore	Switzerland	Brunei Darussalam
Target customers	Employers	Individuals and employers	Individuals, insurers, and employers	Health providers, insurers, and employers	Health providers, public health policymakers, and individuals
Domains of health	BMI, waist circumference, sleep, drinking, and smoking habits	Smartwatch data (physical activity, sleep, nutrition, and stress) and self-logged metrics, such as BP^a^, lipid, HbA_1c_^b^, HRV^c^, HR^d^, SpO2^e^, temperature, sleep, and exercise	Physical activity, sleep, sedentary behavior, nutrition, and sleep	Physical activity, mindfulness, sleep, mental well-being, physical health, nutrition, and self-control	Physical health, mental health, diet, physical activity, and frailty
Data sources	Self-reported data	Wearable devices and self-reported data	Wearable devices and self-reported data	Wearable devices and self-reported data	EMRs^f^, validated questionnaires (DASS-21^g^, SFFFQ^h^, IPAQ-SF^i^, and FRAIL^j^)
Downstream actions	Coaching, rewards, and incentives	Habit-forming programs, digital health coach, and rewards	Challenges	Personalized lifestyle recommendations	Risk screening, personalized lifestyle recommendations, incentives, policymaking, and links to health care services

a BP: blood pressure.

b HbA_1c_: hemoglobin A_1c_.

c HRV: heart rate variability.

d HR: heart rate.

e SpO2: blood oxygen saturation.

f EMR: electronic medical record.

g DASS-21: Depression Anxiety and Stress Scale-21.

h SFFFQ: Short Form Food Frequency Questionnaire.

i IPAQ-SF: International Physical Activity Questionnaire-Short Form.

j FRAIL: Fatigue, Resistance, Ambulation, Illnesses, and Loss of weight.

These existing commercially available health scores mostly target either individual users, employers, or insurers. Most of the products only cover limited domains of health, including a combination of biometric and self-reported data to assess domains, such as physical activity, sleep, nutrition, and mental well-being. Common downstream applications include personalized digital coaching and incentive-based health promotion. Most of the products do not integrate with the national health data infrastructure and do not provide links to health care services.

In addition, most of these health scores are either commercially driven or focused on behavior modification within corporate wellness or insurance settings. In contrast, the Health Index was designed within a public health framework in Brunei Darussalam. It incorporates a wide range of health factors into the computation, and supports a broader range of downstream applications, including risk screening, policymaking, personalized lifestyle recommendations, and health care system connectivity. This positions Health Index as a more holistic and contextually adaptable tool for population-level health monitoring and policy planning.

Furthermore, although these methods were scientifically developed, to the best of our knowledge, there are no relevant scientific publications to show their effectiveness. To assess their scientific validity, we conducted a structured search across gray literature sources, including company websites, product white papers, and regulatory filings where available. Our search found limited evidence of formal validation studies published in the scientific literature. In most cases, the details of the algorithm were proprietary, and there were no rigorous evaluation or clear documentation of the predictive accuracy.

### Objective

Overall, our Health Index research will span over several phases as follows:

1. To design and develop the Health Index — a composite score that measures overall health status across multiple domains.
2. To validate the Health Index at both domain and overall levels, assess responses, individual item scores, domain weights, and cut-off points using appropriate statistical analyses.
3. To create a shorter version of the Health Index that retains strong psychometric properties.
4. To incorporate a machine learning (ML)-based predictive analysis tool into the validated and stable Health Index tool to estimate disease risk and enable timely preventive action.

**Figure 1.**
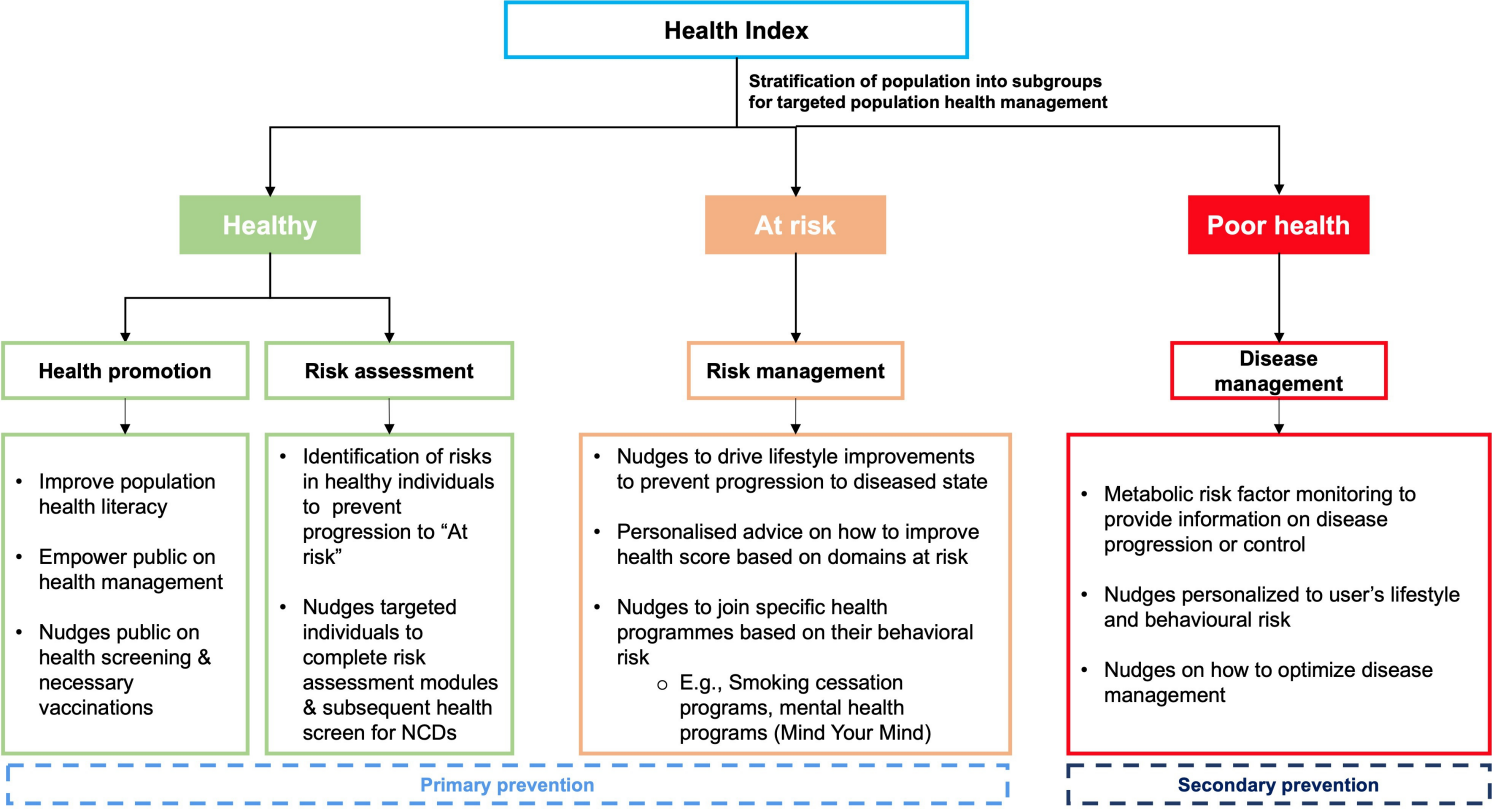
Health Index targeted population health management for healthy, at-risk, and poor health groups. NCD: noncommunicable disease.

In this study, as the first phase, we propose a Health Index to tackle the limitations of existing health scores, which is developed based on the concept of lifestyle medicine. The module would be embedded into the BruHealth app, and users can update their profile and their overall health status using the Health Index. The users will be stratified into either one of healthy, at-risk, or poor health groups for targeted population health management (Figure 1). The objective of this study is to describe the concept of Health Index and its pilot deployment. This pilot implementation focuses on the integration of Health Index into the national mHealth app and the initial rollout to the general population. While statistical evaluation is outside the scope of this study, future phases will scientifically assess the effectiveness of Health Index, describing individuals’ overall health status using health care use data.

## Methods

### Study Design and Participant Flow

This is a prospective observational study conducted through the national BruHealth mobile app launched in 2023. The objective of this study is to describe the concept of Health Index and its pilot deployment. This pilot implementation focuses on the integration of Health Index into the national mHealth app and the initial rollout to the general population.

Apart from describing the concept and pilot implementation, this study also presents the preliminary results derived from 166 early participants to find out the relationship between Health Index results and health care use. In the field study, users were invited to participate through the BruHealth mobile app. After giving consent, participants then proceeded to complete the Health Index questionnaires. The outcome measure, which was used for the preliminary analysis, is the count of NCD-related outpatient visits within 1 year before the completion of the Health Index. This variable was extracted from the national electronic medical record (EMR) system, Brunei Health Information and Management System (Bru-HIMS).

### Users Information

Given that the Health Index is a population health tool and will be available for all accounts with the latest version of BruHealth mobile app, users are invited to participate in the Health Index. Participation is completely voluntary and is open to all BruHealth users who are 18 years and older (which is also the qualifying age for BruHealth). Individuals who are pregnant or aged 17 years and younger are excluded.

### Data Management

**Figure 2.**
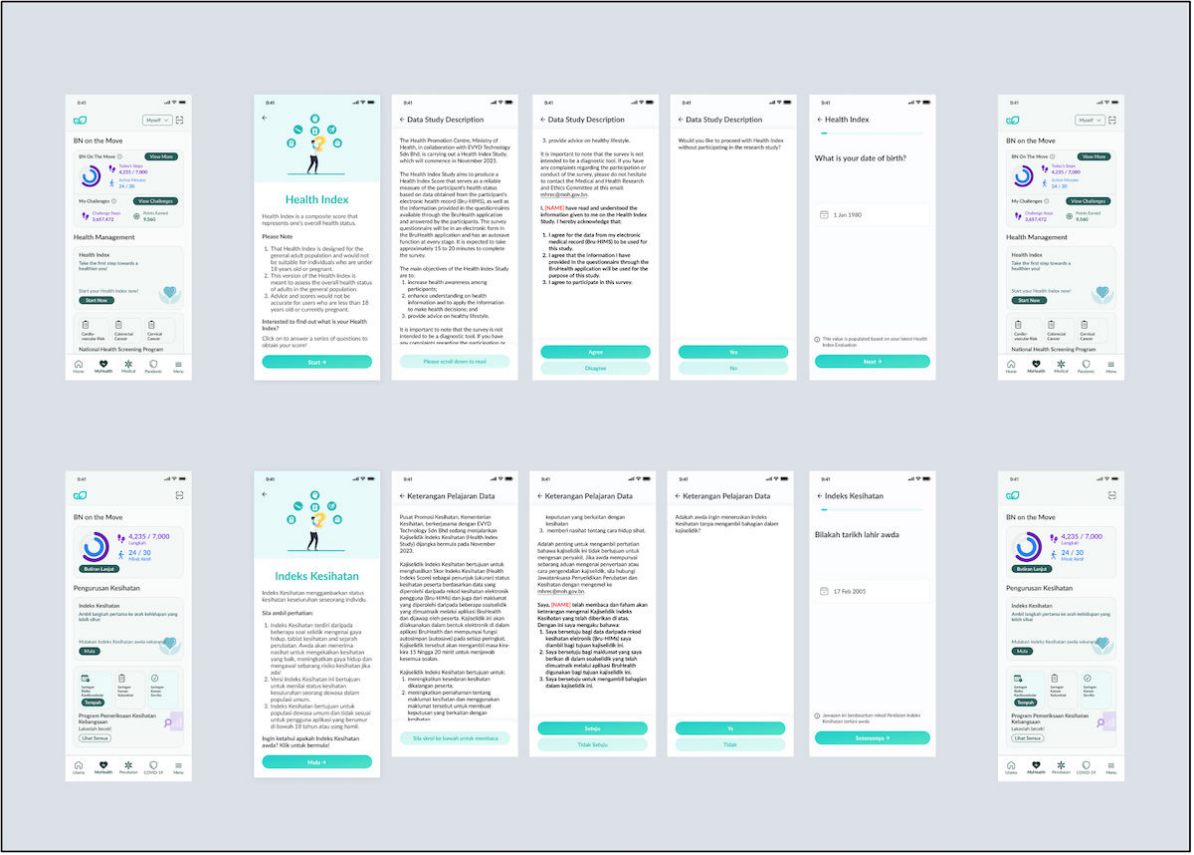
English and Malay user interface of Health Index.

Users of BruHealth will be invited to participate in this validation study. Users will be prompted to take the Health Index either through push notifications, banners, or cards on the BruHealth mobile app, after which they will be directed to a Participant Information Sheet (PIS) and Consent Form (Figure 2). As part of our localization effort, the PIS and Consent Form are also translated into the Malay language. They will be sent 2 types of notifications: (1) for recruitment to participate in the Health Index, and (2) reminders to nudge for completion of questionnaires to get a final Health Index result. Only the first completed data for each participant will be collected for data analysis.

Data for the study will be collected from (1) the national EMR system, Bru-HIMS, and (2) BruHealth (mobile app). Prior to analysis, all records will be desensitized and anonymized by removing personal identifiers (eg, names, identity card numbers, dates of birth). The Ministry of Health, Brunei Darussalam, retains full data ownership and authorizes access only to approved researchers, who are subject to security audits.

The anonymized dataset will be aggregated into data tables and stored in secure servers that meet national data governance and security standards. Data will be stored for 5 years after the completion of the research study and will be destroyed thereafter in compliance with the Ministry of Health’s regulations.

### Structure of Health Index

This study describes the pilot deployment of Health Index, which is integrated into the national BruHealth mobile app. The Health Index comprises 5 domains as follows:

1. Physical health: the physical health domain captures users’ basic profiles, important biomarkers, and lifestyles that are correlated to the development of NCDs.
2. Mental health: as one pillar of lifestyle medicine, mental health problems can lead to several health issues, such as obesity and immune dysfunction.
3. Diet: food intake has a large impact on health. An unhealthy diet, especially low fiber, high sugar, and saturated fat intake, can contribute to the development of NCDs.
  - Physical activity: Physical activity is an area where related literature is abundant and continuously shows its health-promoting benefits. Regular physical activity can result in better psycho-physical health.
4. Frailty: we measure frailty for users older than 60 years as a way to predict the risk of adverse outcomes associated with frailty. Based on the results, specific interventions or treatment plans can be developed.

The selection of health domains included in the Health Index was informed by a combination of literature review and expert consensus. The physical health domain is useful to capture biomarker information that is commonly used by health professionals to identify and manage chronic diseases. Some of the biomarkers asked in Health Index are blood pressure, low-density lipoprotein level, and hemoglobin A_1c_ level, all of which are important biomarkers of metabolic syndrome and other common NCDs. Mental health, diet, and physical activity are 3 of the main pillars of lifestyle medicine, which is a branch of evidence-based health care prioritizing disease prevention rather than treatment [17]. Mental health and stress are known risk factors affecting chronic diseases and mortality [17]. Moreover, multiple studies have confirmed the benefits of a healthy diet on chronic disease prevention and even reversal [17]. Physical activity is another pillar of lifestyle medicine that has a profound impact on longevity and health span [17]. Finally, frailty assessment is useful to identify older adults who are at higher risk of adverse health outcomes.

**Figure 3.**
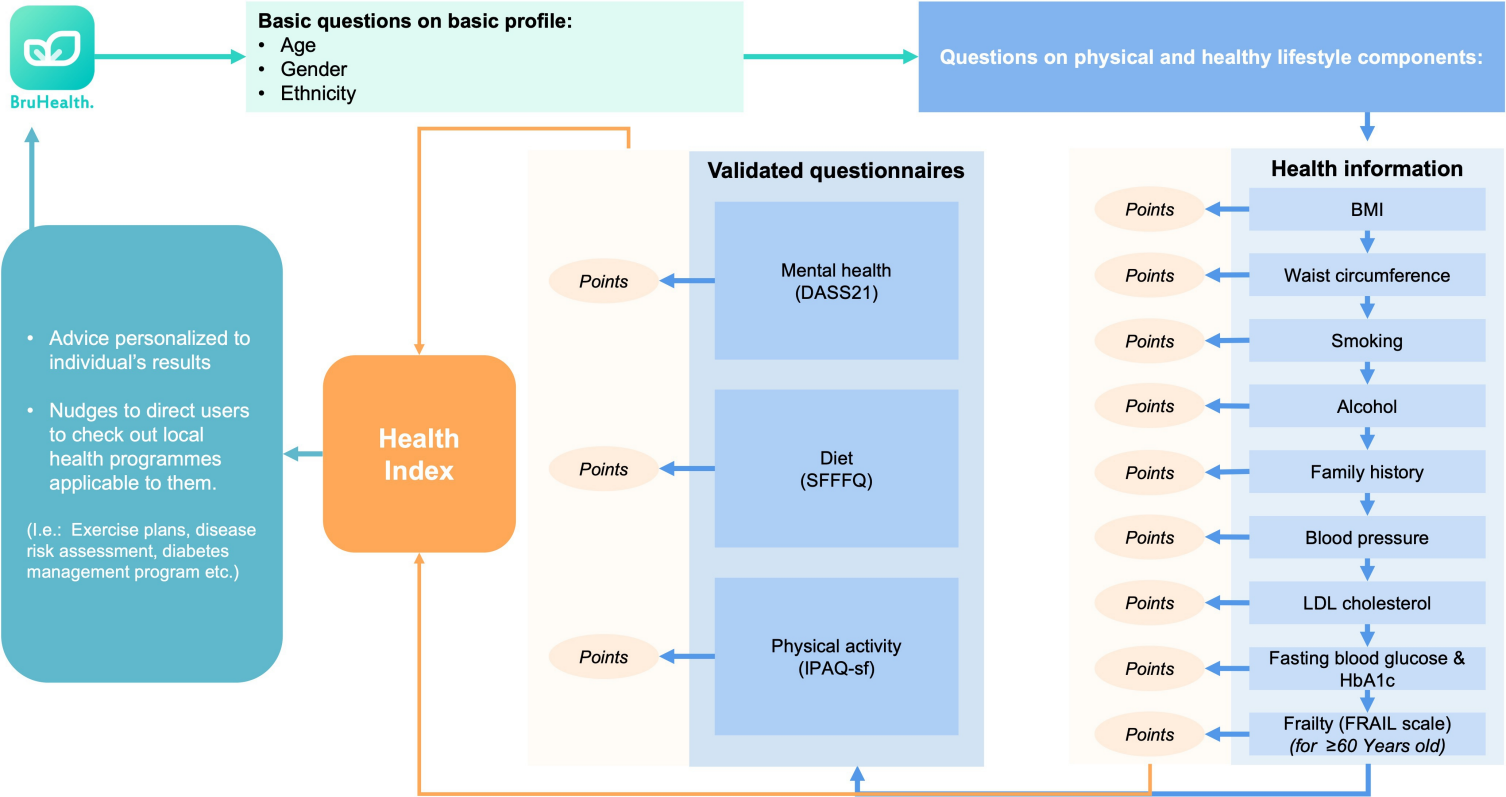
Health Index user workflow. DASS-21: Depression Anxiety and Stress Scale 21; FRAIL: Fatigue, Resistance, Ambulation, Illnesses, and Loss of weight; HbA_1c_: hemoglobin A_1c_; IPAQ-SF: International Physical Activity Questionnaire-Short Form; LDL: low-density lipoprotein; SFFFQ: Short Form Food Frequency Questionnaire.

Once entering the Health Index page, users’ health profiles will be created, and the overall health status will be calculated based on 3 main sources of data, including (1) EMR, (2) self-reported data, and (3) validated questionnaires on mental health, diet quality, physical activity, and frailty. First, users will be asked a series of basic demographic questions followed by physical health and lifestyle questions. These include biochemical lab tests that have been conducted, as well as family history and past medical history (Figure 3). Basic demographic data and the latest available biochemical data (defined as relevant tests conducted within the past 1 year prior to filling in the Health Index), if available, will be extracted from Bru-HIMS and auto-populated in the respective questions. Table 2 lists all the questions that capture this kind of information.

**Table 2.** List of questions to capture users’ physical health and lifestyles.

Domains	Questions
Basic profile	What is your date of birth? What is your birth gender? What is your ethnicity?
Physical health	What is your height? What is your current weight? What is your most recent waist circumference?
Lifestyle	What is your smoking status? Do you drink alcohol? On average, how many standard drinks do you drink a week? (Regular beer: 1 can, 330 ml; hard liquor: 1 shot, 30 ml; wine: 1 small glass, 100 ml)
Basic profile	Do you have any family members who are diagnosed with any of the following? Diabetes mellitus First-degree family member with premature CVD^a^ (male <55 y, and female <65 y) Familial hyperlipidemia (genetic condition that leads to high cholesterol levels)
	Do you have any medical history of the following conditions: CVDs Cerebrovascular diseases Cancer Hypertension Diabetes Unsure
Physical health	What is your most recent systolic blood pressure (the upper reading on monitor)? What is your most recent diastolic blood pressure (the lower reading on monitor)? Have you ever been diagnosed with hypertension (high blood pressure) before? What is your most recent LDL^b^ cholesterol level (bad cholesterol)? Have you ever been diagnosed with dyslipidemia (high cholesterol) before? Have you ever been diagnosed with type 2 diabetes mellitus before? What is your most recent fasting blood glucose level? What is your most recent HbA_1c^c^_ level?

a CVD: cardiovascular disease.

b LDL: low-density lipoprotein.

c HBA_1c_: hemoglobin A_1c_

Bru-HIMS is a national e-health initiative that was launched by the Ministry of Health in September 2012 [18]. The initiative adopts the concept of “One Patient, One Health Record,” where each individual’s health care data from all government hospitals, outpatient services, treatment centers, and clinics is together under one EMR. This will allow health care professionals access to patient records in real time at the point of care throughout government hospitals and health centers nationwide.

Next, users will be asked to complete a series of questionnaires for objective assessment. These questionnaires are used to assess users’ mental health status, diet quality, and physical activity level. These questionnaires include the Depression Anxiety and Stress Scale 21 (DASS-21) for emotional states of depression [19], anxiety, and stress; the Short Form Food Frequency Questionnaire (SFFFQ) for diet quality [20]; and the International Physical Activity Questionnaire-Short Form (IPAQ-SF) to measure physical activity level [21]. For users aged 60 years and above, an additional questionnaire will be included to screen for frailty. The validated Fatigue, Resistance, Ambulation, Illnesses, and Loss of Weight (FRAIL) scale questionnaire is used for this purpose [22]. We have obtained approvals from relevant bodies to use these validated questionnaires in the Health Index.

The Health Index scores will then be calculated using all 13 factors of health criteria mentioned above. It is computed using a multidomain, rule-based scoring framework that integrates all the 13 health factors. While the exact algorithm and weightages are proprietary and cannot be publicly disclosed, the scoring system, including weightages, was designed with input from a multidisciplinary panel of local medical domain experts based in primary health care, community nutrition, sports and exercise medicine, psychiatry and psychology, geriatrics, as well as health promotion. The scoring methodology is as follows:

*HI = S_i_* [*physical_health* x *w*_*1*_ *+ mental_health* x *w*_*2*_ *+ diet* x *w*_*3*_ *+ physical_activity* x *w*_*4*_ *+ frailty* x *w*_*5*_ ]

where *HI* is Health Index; *S_i_* is normalized score (0 to 100 scale with min-max normalization); *w_i_* are weights for each domain (for users under 60 y, the weight for frailty is 0 and the weights are redistributed proportionally among the other 4 domains). In the current Health Index, we apply equal weightages (*w_i_*) for all 5 health domains. However, as we gather more data, we aim to recompute the weightages by applying various statistical techniques, such as the graded response model (of item response theory), and ML techniques in the future.

A scoreboard system is used to map the severity of risk factors to derive the final numerical score, which is a number between 0 and 100. No normalization is performed, and the raw value is used for mapping. The panel considered the relative impact of each factor on overall health, based on both clinical experience and literature review. The scores for each health domain are derived through appropriate guidelines from different validated sources (Table 3). Future research will focus on validating the algorithm’s predictive performance and real-world use. Future versions of Health Index will explore the application of ML techniques to replace the fixed, arbitrary, and rule-based scoring systems.

**Table 3.** Scores for health domains based on different guidelines.

Health domains	Guidelines and their references
BMI	WHO^a^ [23,24]
Waist circumference	WHO [24]
Blood biomarkers	Brunei Darussalam National Hypertension Guideline [25] Brunei Darussalam National Hyperlipidaemia Guidelines [26] Ministry of Health Singapore Clinical Practice Guidelines: Lipids [27] Brunei Darussalam National Health Screening Guideline on NCDs^b^ 2020 [28]
Smoking	WHO [29]
Alcohol	The American College of Cardiology/American Heart Association Guideline [30]

a WHO: World Health Organization.

b NCD: noncommunicable disease.

However, while Health Index gives a more comprehensive view of an individual’s overall health status, it is important to acknowledge the limitations of the data sources. Self-reported data are often subject to recall bias, social desirability bias, and misreporting, which can affect the reliability of inputs such as lifestyle behaviors or perceived health conditions. Similarly, EMRs may vary in completeness and accuracy due to inconsistent documentation practices, missing data, or differences in coding standards. All these limitations may present uncertainty in the calculation of the Health Index and should be considered when interpreting results.

Finally, a Health Index result will be generated to assess users’ health status, categorizing them as either healthy, at risk, or in poor health (Textbox 1). The preliminary risk classification thresholds based on expert consensus are as follows:

Textbox 1. **A sample of description of the “At Risk” category.**

Your result places you in the “At Risk” category. You may have certain risk factors that may contribute to poor health outcomes in the future. To find out more about how you can improve your overall health and reduce your risk of developing chronic diseases, tap on the cards under health advice.

Disclaimer: To improve the accuracy of your score, do ensure that you completed all questions with the most accurate information.

1. Healthy: score ≥82 indicates minimal risk. The cutoff equals the total of each health domain’s low-risk minimum score multiplied by its weightage.
2. At risk: scores between 31 and 81 indicate moderate risk requiring intervention. The cutoff is calculated by multiplying the minimum and maximum scores of the moderate-risk categories in each health domain by their respective weightages and then summing the results.
3. Poor health: score ≤30 indicates significant risk. The cutoff is determined by summing the weighted maximum scores of the high-risk categories across all health domains.

**Figure 4.**
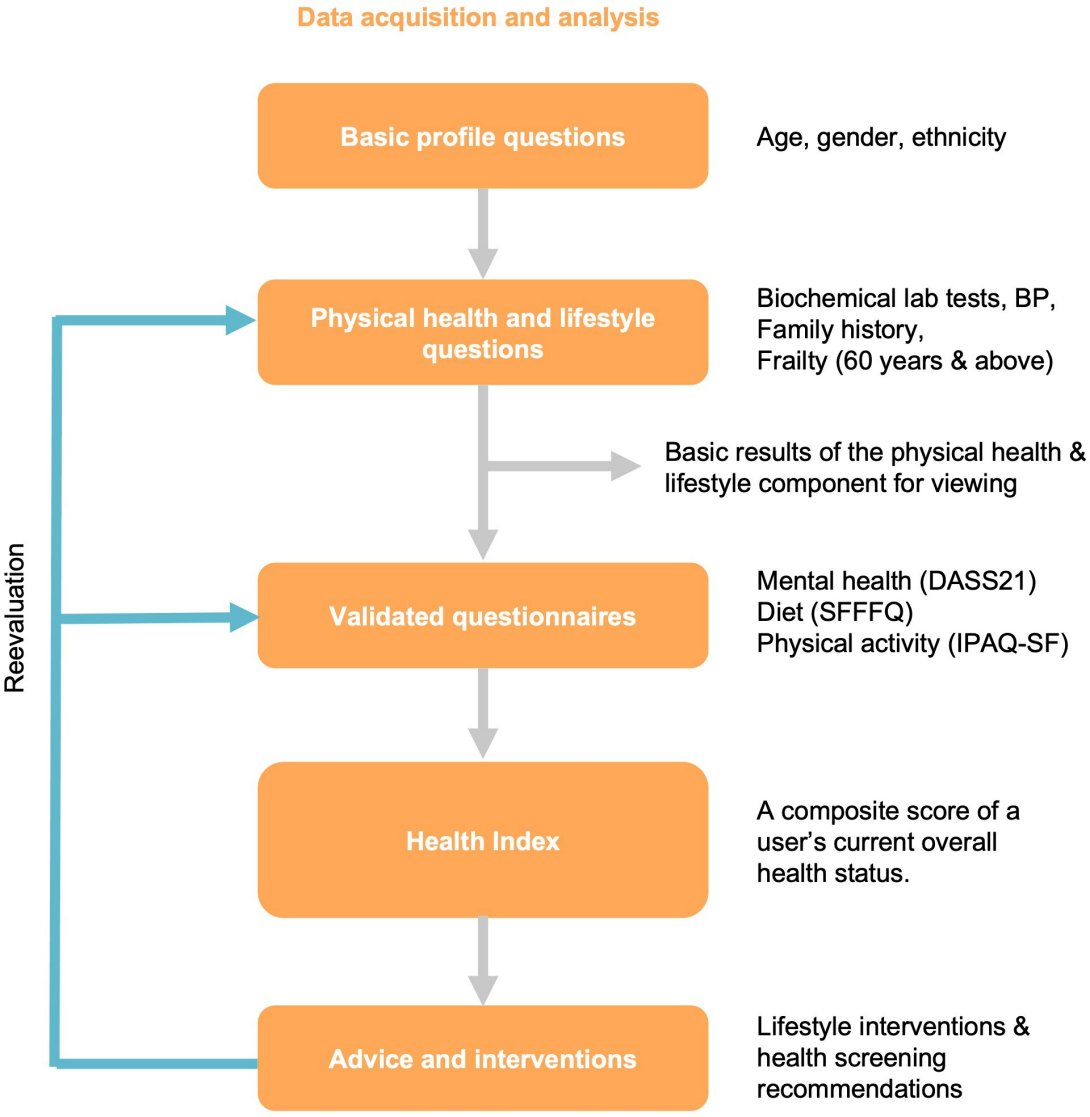
General flow of Health Index. BP: blood pressure; DASS-21: Depression Anxiety and Stress Scale 21; IPAQ-SF: International Physical Activity Questionnaire-Short Form; SFFFQ: Short Form Food Frequency Questionnaire.

Users will also be able to view health advice based on their answers to the questionnaires (Textbox 2). Figure 4 depicts the general flow of the Health Index model.

Textbox 2. **A sample of health advice that will be received by users.**

Health domain (BMI) and Score (at risk), overweight (≤29.9 kg/m^2^)

Health advice: your BMI is in the overweight category. Being overweight puts you at risk of developing chronic diseases such as diabetes, hypertension, and coronary heart disease. Start adopting a healthy and active lifestyle before it’s too late! Lowering your BMI starts with increasing your physical activity and sticking to a healthy eating plan.

### Data Analysis and Validation Strategy

The Health Index is intended to undergo future validation using appropriate statistical methods to assess its reliability. These validation efforts will include assessing the potential associations with clinical outcomes using real-world health data.

The objective of the data analysis is to retrospectively investigate if the Health Index score is correlated with participants’ actual health outcomes using the existing data in Bru-HIMS. The health outcomes of interest are NCDs, including but not limited to diabetes, ischemic heart disease, stroke, chronic kidney disease, breast cancer, colorectal cancer, chronic obstructive pulmonary disease, lung cancer, and other morbidities. We consider NCD-related health care use, which includes length of hospital stays, number of visits to primary care centers, as well as hospitals, and the medication prescription status. To note, health care use has been used as a target variable when validating risk prediction tools [31-33].

To validate the Health Index framework, several statistical tests will be conducted to examine whether there are statistically significant differences in the NCD-related health care use among participants with different Health Index scores and categories. As we would only use the data for participants who have completed the whole Health Index questionnaire, missing data are not expected in the study. First, differences in health care use across different Health Index categories (“healthy,” “at risk,” and “poor health”) will be assessed using one-way ANOVA or Kruskal-Wallis tests. Next, we could investigate if participants in 3 different health categories have a significantly different ratio of individuals with morbidities using a chi-square test. Moreover, the correlation between the Health Index raw score and health care use can be assessed using the Pearson correlation coefficient. Stratification may be done to control for potential confounding factors, including age, sex, and comorbidities. The receiver operating characteristic analysis will be used to evaluate Health Index’s discriminative ability for predicting NCD diagnoses. Sensitivity and specificity metrics will be used to assess whether Health Index performs as indicated by cross-validation with existing NCD diagnoses in predicting NCD outcomes and health care use. Exploratory factor analysis (EFA) and subsequent confirmatory factor analysis (CFA) will be used to assess convergent and divergent (discriminant) validity. EFA will be used to explore the underlying items of Health Index’s 5 domains to ensure that items load primarily on their intended domains with minimal cross-loadings. Whereas, CFA will confirm the factor structure, using fit indices, of the domains in the survey as a whole.

In addition to the above analysis, an assessment of the Health Index test–retest reliability is also planned. A subset of consenting participants may be invited to repeat the Health Index assessment after a defined time interval (eg, 2 weeks), enabling evaluation of temporal consistency using intraclass correlation coefficients or Cohen kappa.

Furthermore, to assess the initial scoring and weights mentioned above and move from expert consensus to data-driven weightages, we will use the graded response model (of item response theory), EFA, and CFA, structural equation modeling for multivariable outcomes, using clinical outcome variables as a criterion reference. In addition to receiver operating characteristic analysis, regression-based cutpoint estimation, and change-point or segmented regression models will be used to establish the cut-off points for risk levels.

Using G*Power software (Heinrich Heine University) [34], we calculated the sample size needed using the following parameters: (1) effect size=0.3, (2) α=.05, and (3) power=0.80. Based on these inputs, it estimated a total sample size of at least 36 participants per Health Index category needed.

### Ethical Considerations

This study was reviewed and approved by the Medical and Health Research and Ethics Committee of the Brunei Darussalam Ministry of Health, under approval reference number MHREC/MOH/2023/22(1). All participants provided informed consent electronically within the BruHealth mobile app. A consent form has been obtained for all participants, and no financial compensation was given for taking part in this study. All data collected were stored in safe, password-protected computers, which were only accessible to the researchers. All data were de-sensitised and anonymised prior to analyses by removing all personal identifiable information such as names, identity card numbers and dates of birth.

## Results

**Figure 5.**
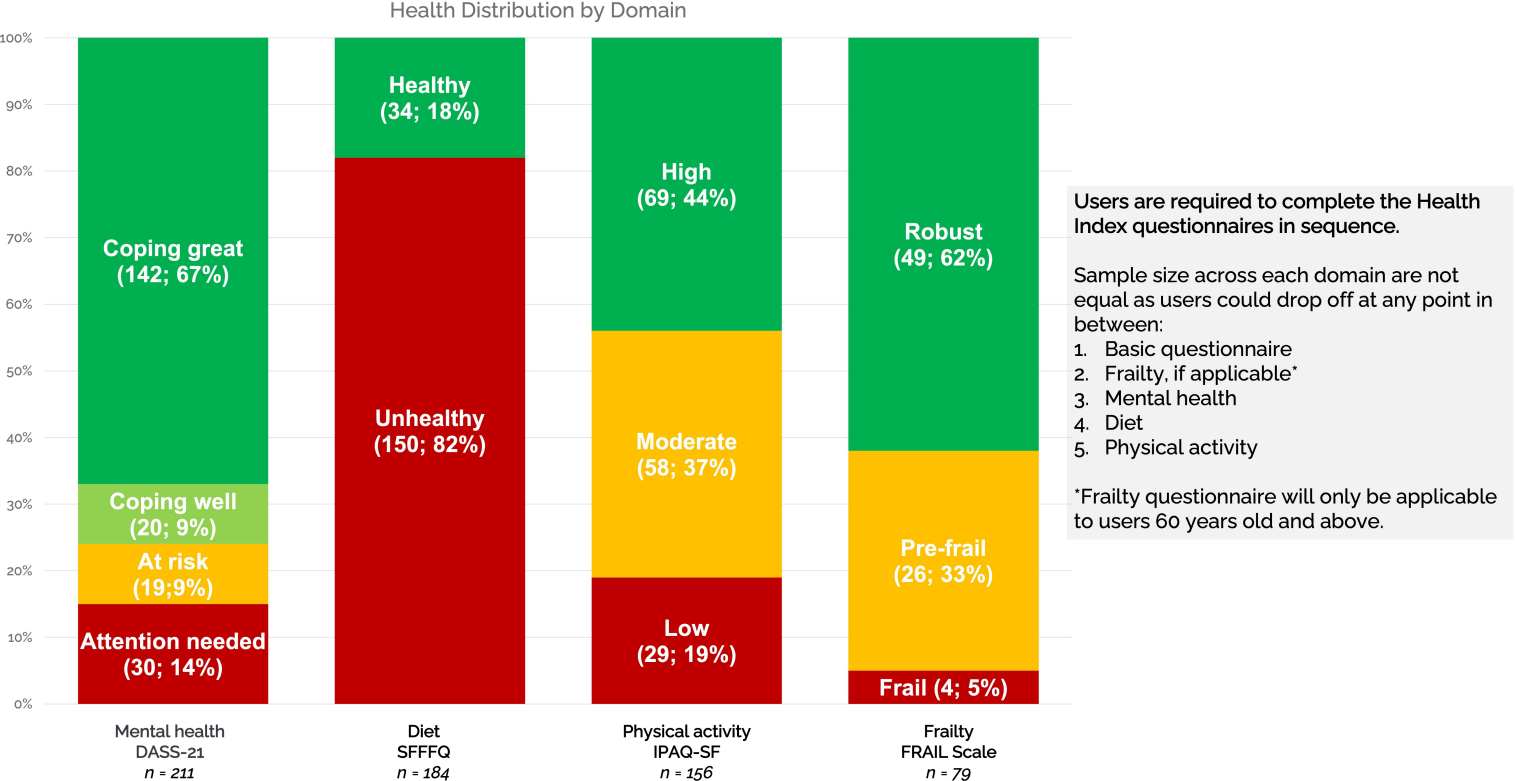
Unhealthy diet as the most prominent outcome of potential concern. DASS-21: Depression Anxiety and Stress Scale 21; FRAIL: Fatigue, Resistance, Ambulation, Illnesses, and Loss of Weight; IPAQ-SF: International Physical Activity Questionnaire-Short Form; SFFFQ: Short Form Food Frequency Questionnaire.

Previously, a preliminary national field test for Health Index was conducted to obtain health-seeking behavioral insights on the Health Index use. A total of 1200 active users on BruHealth were randomly selected according to age and gender. A total of 13.8% (166/1200) of the targeted users completed the questionnaires within 1 month. One of the main findings was 85% (1019/1200) of the participants were in the “At Risk” category, 13.9% (167/1200) of them were in the “Healthy” category, and 1.2% (14/1200) of them were in the “Poor Health” category. Another interesting finding is that diet was the most prominent outcome of potential concern (Figure 5).

The median age of the participants was 45 (interquartile range (IQR) 36‐58) years. The female-to-male ratio was 53% (636/1200): 47% (564/1200). In terms of ethnicity, 83.1% (997/1200) were Malays, 11.5% (138/1200) Chinese, and 5.4% (65/1200) from other ethnic groups. The distribution of participants across districts was as follows: 75.9% (911/1200) from Brunei Muara, 15.7% (188/1200) from Belait, 7.2% (86/1200) from Tutong, and 1.2% (14/1200) from Temburong. These figures are similar to the population figures provided by the Department of Economic Planning and Statistics, Ministry of Finance and Economy, Brunei Darussalam [35].

**Figure 6.**
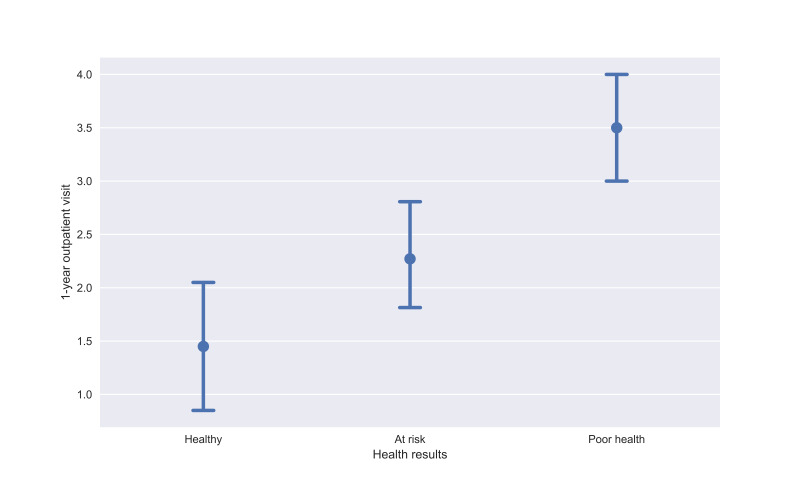
Noncommunicable disease–related health care use among different Health Index categories.

Furthermore, we tried to briefly analyze the correlation between Health Index and NCD-related health care use as the health care outcome, which is the number of outpatient visits related to NCDs in the past 1 year (Figure 6). In this preliminary analysis, we identify differences between different health categories in terms of the counts of outpatient visits. Those people in at-risk and poor health groups tend to have higher NCD-related health care use compared to those in the healthy group. However, we acknowledge that the results are preliminary, and we have a small sample size as well as group imbalance. Therefore, we are committed to continuing to validate our Health Index with a larger sample size in the future.

With effective nudging strategies, which can potentially increase public interest in participation, the Health Index is able to capture a holistic health status of participants while considering existing diagnoses, showing great promise as a population health management tool.

## Discussion

### Principal Findings

In this study, we present the initial user engagement to assess its feasibility for large-scale deployment, laying the groundwork for future validation studies. Health Index can improve early identification of individuals at risk of developing NCDs and support health promotion through personalized feedback, thus contributing to more targeted and effective prevention strategies. Long-term goals include improving health literacy, promoting the adoption of healthier behaviors, and enabling earlier identification of individuals at risk for NCDs. These outcomes will be measured using follow-up user surveys, behavioral tracking, and linkage to health care use data. As discussed above, we acknowledge several limitations of the Health Index in its current form, including its reliance on self-reported data, which are often subject to bias. To address this, we plan to enable users to give permission for the app to extract data from their EMR stored in the Bru-HIMS to complete several physical health-related questions or use wearable devices to answer lifestyle-related questions. Apart from that, we plan to perform a reliability analysis to measure the consistency of the Health Index when repeated several times within a short period. Another limitation is the arbitrary thresholds and small pilot sample size. To tackle these issues, we plan to perform a graded response model, EFA, and CFA, and apply ML models to assess the initial scoring and weights. As the sample size would continuously increase, we would repeat our validation approaches to revalidate the Health Index on a larger sample size. Furthermore, we recognize the potential of ML to improve accuracy and personalization in future iterations. At this stage, the integration of ML has not been implemented or evaluated in the current version of Health Index. In the future, we plan to incorporate ML into the Health Index. The Health Index framework will be improved by identifying any clinical factors (eg, lab tests, demographics, family history, etc) based on Brunei population data that are associated with good prediction of the health outcomes of interest, which have not been identified in the current framework.

One possibility is to use ML to calculate the importance of each domain in the Health Index. One popular method to apply is the Shapley additive explanations (SHAP) method [36], which is a game theory-based method for explaining the results generated by any ML model. Based on the weights derived from SHAP, the weights of the domains in Health Index can be adjusted to better capture the effects of specific factors on users’ health care outcomes. Not only can the domain weights be computed, but also the weight for each of the questions in the questionnaires if sufficient data can be collected. Different populations may have different magnitudes of importance of factors that affect the health care outcomes. Through weight adjustment, the Health Index can be continuously improved to meet the needs of different populations with different characteristics.

Another possibility where ML can play an important role in the Health Index is dimensionality reduction or feature selection. In the future, we aim to reduce the number of questions to improve the user experience. To implement this, several ML models can be used. First, SHAP could still be applied to find out the importance of each question in the questionnaires. Then, top *k* questions can be chosen based on the importance, while the rest can be removed. Other ML techniques that can be used for this purpose are information gain, Fisher score, and wrapper methods.

### Conclusions

NCDs typically develop over many years and cause symptoms. Therefore, identifying the risk factors and addressing them early is the most cost-effective solution for public health. In this study, we have proposed a simple yet practical composite health score called “Health Index.” From a health perspective, an increasingly robust Health Index over time is intended for a timelier prompt of NCDs risk assessment for users, which in turn will allow early detection of the key risk factors of NCDs for early actions and health risk management. Resource-wise, findings from the Health Index can support policymakers in planning and allocating appropriate and adequate efforts and resources more effectively in the country. Identified risk factors can also direct national health plans and strategies in Brunei Darussalam. Finally, it can also support policymakers in setting outcome-based measures to drive overall quality and value improvements. In the future, we aim to retrospectively and prospectively validate the Health Index, as well as address some of its limitations as discussed previously.

## Abbreviations

Bru-HIMS: Brunei Health Information and Management System
CDC: Center for Disease Control and Prevention
CFA: confirmatory factor analysis
CVD: cardiovascular disease
DASS-21: Depression Anxiety and Stress Scale 21
EFA: exploratory factor analysis
EMR: electronic medical record
FRAIL: Fatigue, Resistance, Ambulation, Illnesses, and Loss of Weight
IPAQ-SF: International Physical Activity Questionnaire-Short Form
LWB-I: lifestyle and well-being index
mHealth: mobile health
ML: machine learning
NCD: noncommunicable disease
PIS: participant information sheet
SFFFQ: Short Form Food Frequency Questionnaire
SHAP: Shapley additive explanations
WHO: World Health Organization

## References

[1] (2022). Noncommunicable diseases progress monitor 2022. https://www.who.int/publications/i/item/9789240047761.

[2] (2021). Brunéi Darussalam multisectoral action plan for the prevention and control of noncommunicable diseases BruMAP-NCD 2021-2025. https://moh.gov.bn/wp-content/uploads/2024/10/BRUMAP-NCD-2021_compressed.pdf.

[3] Naghavi M, Ong KL, Aali A (2024). Global burden of 288 causes of death and life expectancy decomposition in 204 countries and territories and 811 subnational locations, 1990-2021: a systematic analysis for the Global Burden of Disease Study 2021. Lancet.

[4] Noncommunicable diseases fact sheet. World Health Organization.

[5] Ferrari AJ, Santomauro DF, Aali A (2024). Global incidence, prevalence, years lived with disability (YLDs), disability-adjusted life-years (DALYs), and healthy life expectancy (HALE) for 371 diseases and injuries in 204 countries and territories and 811 subnational locations, 1990-2021: a systematic analysis for the Global Burden of Disease Study 2021. Lancet.

[6] Goel S, Verma M, Mohapatra A, Popovic S (2025). Editorial: Raising awareness around trends in noncommunicable diseases and their risk factors to promote global prevention and control. Front Public Health.

[7] Pano O, Sayón-Orea C, Hershey MS, Bes-Rastrollo M, Martínez-González MA, Martínez JA (2022). Development of a general health score based on 12 objective metabolic and lifestyle items: the lifestyle and well-being index. Healthcare (Basel).

[8] Employee health. TELUS Health.

[9] ConnectedLife.

[10] Digital health. Aktivo Labs.

[11] Dynamic health scoring technology for businesses. Dacadoo Health Score.

[12] Koh KS, Lim HS, Lim J, Wei Y, Minn PW, Wong J (2022). Development and implementation of a national mobile health application: a case study from Brunei. J Glob Health.

[13] Stone NJ, Robinson JG, Lichtenstein AH (2014). 2013 ACC/AHA guideline on the treatment of blood cholesterol to reduce atherosclerotic cardiovascular risk in adults: a report of the American College of Cardiology/American Heart Association task force on practice guidelines. Circulation.

[14] Tangri N, Stevens LA, Griffith J (2011). A predictive model for progression of chronic kidney disease to kidney failure. JAMA.

[15] Gail MH, Brinton LA, Byar DP (1989). Projecting individualized probabilities of developing breast cancer for white females who are being examined annually. J Natl Cancer Inst.

[16] Luu HN, Behari J, Goh GBB (2021). Composite score of healthy lifestyle factors and risk of hepatocellular carcinoma: findings from a prospective cohort study. Cancer Epidemiol Biomarkers Prev.

[17] Lippman D, Stump M, Veazey E (2024). Foundations of lifestyle medicine and its evolution. Mayo Clin Proc Innov Qual Outcomes.

[18] Bru-HIMS: overview. Ministry of Health.

[19] Lovibond SH, Lovibond PF (1995). Manual for the Depression Anxiety Stress Scales (DASS--21, DASS--42) [Database Record.

[20] Persson CE, Rothenberg E, Hansson PO, Welin C, Strandhagen E (2019). Cardiovascular risk factors in relation to dietary patterns in 50-year-old men and women: a feasibility study of a short FFQ. Public Health Nutr.

[21] van Poppel MNM, Chinapaw MJM, Mokkink LB, van Mechelen W, Terwee CB (2010). Physical activity questionnaires for adults: a systematic review of measurement properties. Sports Med.

[22] Woo J, Yu R, Wong M, Yeung F, Wong M, Lum C (2015). Frailty screening in the community using the FRAIL Scale. J Am Med Dir Assoc.

[23] (1995). Physical status: the use of and interpretation of anthropometry, report of a WHO expert committee. https://www.who.int/publications/i/item/9241208546.

[24] (2008). Waist circumference and waist-hip ratio: report of a WHO expert consultation. https://www.who.int/publications/i/item/9789241501491.

[25] (2019). Brunei Darussalam national hypertension guideline 2019. https://cardiacsociety.org.bn/wp-content/uploads/2019/10/National-Hypertension-Guidelines.pdf.

[26] (2022). Brunei Darussalam national hyperlipidaemia guidelines 2022. Cardiac Society Brunei Darussalam.

[27] (2016). MOH clinical practice guidelines: lipids. Ministry of Health Singapore.

[28] (2021). Brunei Darussalam multisectoral action plan for the prevention and control of noncommunicable diseases (brumap-NCD) 2021-2025. Ministry of Health Brunei Darussalam.

[29] (2019). Tobacco questions for surveys of youth (TQS-youth): a subset of key questions from the global youth tobacco survey (GYTS). https://cdn.who.int/media/docs/default-source/ncds/ncd-surveillance/final_english_tqsyouth_190522.pdf?sfvrsn=b1cd010f_1.

[30] Arnett DK, Blumenthal RS, Albert MA (2019). 2019 ACC/AHA guideline on the primary prevention of cardiovascular disease: a report of the American College of Cardiology/American Heart Association task force on clinical practice guidelines. Circulation.

[31] Balqis-Ali NZ, Jawahir S, Chan YM (2024). The impact of long-term care interventions on healthcare utilisation among older persons: a scoping review of reviews. BMC Geriatr.

[32] Wang M, Shui AM, Ruck J (2024). The liver frailty index is a predictor of healthcare utilization after liver transplantation in older adults. Clin Transplant.

[33] Dolja-Gore X, Byles JE, Tavener MA (2021). Estimating the effect of health assessments on mortality, physical functioning and health care utilisation for women aged 75 years and older. PLoS ONE.

[34] Faul F, Erdfelder E, Lang AG, Buchner A (2007). G*Power 3: a flexible statistical power analysis program for the social, behavioral, and biomedical sciences. Behav Res Methods.

[35] (2024). Brunei Darussalam key indicators 2024. Department of Economic Planning and Statistics, Ministry of Finance and Economy, Brunei Darussalam.

[36] Lundberg SM, Lee SI (2017). A unified approach to interpreting model predictions. arXiv.

